# The lack of association between the burden of monosodium urate crystals assessed with dual-energy computed tomography or ultrasonography with cardiovascular risk in the commonly high-risk gout patient

**DOI:** 10.1186/s13075-018-1602-3

**Published:** 2018-05-29

**Authors:** Tristan Pascart, Benoist Capon, Agathe Grandjean, Julie Legrand, Nasser Namane, Vincent Ducoulombier, Marguerite Motte, Marie Vandecandelaere, Hélène Luraschi, Catherine Godart, Eric Houvenagel, Laurène Norberciak, Jean-François Budzik

**Affiliations:** 1 Department of Rheumatology, Lille Catholic Hospitals, University of Lille, F-59160 Lomme, France; 2 Department of Radiology, Lille Catholic Hospitals, University of Lille, F-59160 Lomme, France; 3 Department of Medical Research, Biostatistics, Lille Catholic Hospitals, University of Lille, F-59160 Lomme, France; 4 EA 4490, PMOI, Physiopathologie des Maladies Osseuses Inflammatoires, University of Lille, F-59000 Lille, France; 5 Saint-Philibert Hospital, Rue du Grand But, 59160 Lomme, France

**Keywords:** Dual-energy computed tomography, Ultrasonography, Gout, Cardiovascular risk, Triglycerides

## Abstract

**Background:**

Gout is associated with higher cardiovascular risk that increases with disease severity. The objective of this study was to explore the relationship between the extent of monosodium urate (MSU) crystal deposition, assessed with ultrasonography (US) and dual-energy computed tomography (DECT), and cardiovascular risk.

**Methods:**

Gout patients were included in this cross-sectional study to undergo DECT scans for the assessment of total MSU volume deposition in the knees and feet, and US to evaluate the number of joints with the double contour (DC) sign. Participants were screened for traditional cardiovascular risk factors, and levels of the American College of Cardiology (ACC)/American Heart Association (AHA) 10-year risk for heart disease or stroke were calculated. The primary endpoint was the Spearman correlation coefficient ρ between DECT MSU volume and cardiovascular risk.

**Results:**

A total of 42 patients were included; they were predominantly male (40/42) and aged 63.0 ± 13.2 years. Overall, 28/42 patients presented with the metabolic syndrome and the average 10-year coronary event or stroke risk according to the ACC/AHA (*n* = 33) was 21 ± 15%. Correlations between DECT volumes of MSU deposits in the knees, feet, and knees + feet and cardiovascular risk according to the ACC/AHA were very poor, with ρ = 0.18, −0.01, and 0.13, respectively. The was no correlation between the number of joints with the DC sign and cardiovascular risk (ρ = −0.07). DECT MSU deposit volume was similar in patients with and without metabolic syndrome (*p* = 0.29).

**Conclusions:**

The extent of MSU burden does not increase the estimated risk of cardiovascular events in gout patients.

## Background

Gout is the result of an inflammatory response to monosodium urate (MSU) crystal deposition following prolonged hyperuricemia [1]. The association of gout with increased cardiovascular risk is now fully recognized, but mechanisms linking the two remain unclear [2–4]. Cardiovascular risk in gout seems to increase with disease severity, the presence of clinical tophi, and serum urate (SU) levels [5]. It has been hypothesized that a greater urate load could explain this increased cardiovascular mortality [5]. Systematic evaluation of the cardiovascular risk of gout patients at the time of the diagnosis revealed that a majority of patients are classified as having a high cardiovascular risk [6]. In non-gout patients, SU levels may be associated with higher cardiovascular risk scores but causality of hyperuricemia on cardiovascular comorbidities and events, and the metabolic syndrome, remains uncertain [3, 7]. Furthermore, the association between MSU crystal burden and traditional cardiovascular risk factors needs to be studied. Were they to be correlated, quantifying MSU deposition could help identify gout patients at high cardiovascular risk.

Ultrasonography (US) and dual-energy computed tomography (DECT) are two imaging techniques that can visualize and provide a quantification of the MSU burden [8]. DECT uses two x-ray beams with two different energies allowing us to distinguish between urate and calcium in soft tissues surrounding bone when the volume of deposits exceeds 0.01 cm^3^ [9, 10]. US, on the other hand, can identify intra-articular cartilage MSU deposition appearing as a double contour (DC) sign which disappears during urate depletion [11]. Both techniques can quantify tophi volume but do not provide the same measurements [8].

The main objective of this study was to explore the relationship between the extent of MSU deposition, assessed with US and DECT, and cardiovascular risk assessment.

## Methods

### Patients

Consecutive patients with a diagnosis of gout according to the American College of Rheumatology (ACR)/European League Against Rheumatism (EULAR) 2015 criteria [12] were prospectively recruited to undergo a quantification of urate deposition in the knees and feet using US and DECT [8], and an assessment of their cardiovascular risk. The study was approved by the institutional review board of the Lille Catholic Hospitals and all participants provided informed consent before inclusion into the study.

At the initial clinical visit, the following were recorded: demographic details, comorbid disorders (particularly prior major cardiovascular events), gout history, medications, and physical examination (including measures of body mass index (BMI) and arterial blood pressure (BP)). Laboratory testing of SU levels, lipid levels, blood glucose, and the estimated glomerular filtration rate (eGFR) measured by CKD-EPI or MDRD was to be performed within the 2 weeks of US and DECT examinations.

### Cardiovascular risk assessment and the metabolic syndrome

Traditional cardiovascular risk factors were systematically assessed: BP, BMI, blood glucose, total cholesterol, high-density lipoprotein (HDL) cholesterol, calculated low-density lipoprotein (LDL) cholesterol, triglycerides, and smoking status. Cardiovascular risk was then calculated using the American College of Cardiology (ACC)/American Heart Association (AHA) guidelines [13], the Framingham general 10-year cardiovascular risk factors [14], and the Framingham 10-year risk of coronary disease [15]. These scores could not be calculated for patients whose age was outside the respective range of applicability and for participants with a prior history of coronary heart disease, peripheral arterial disease, and stroke. The metabolic syndrome was defined by the presence of three out of five items among the following: obesity (BMI > 30 kg/m^2^ in the absence of available waist circumference measurements), elevated BP (systolic BP ≥ 130 mmHg or diastolic BP ≥ 85 mmHg or on-going antihypertensive therapy), elevated triglycerides (≥ 15 mg/dL or on-going treatment), low HDL cholesterol (≤ 40 mg/dL in men and ≤ 50 mg/dL in women or on-going treatment), and hyperglycemia (≥ 100 mg/dL or drug treatment for elevated blood glucose) [16].

### US examination

Examinations were performed by one of four trained musculoskeletal radiologists (JFB, NN, BC, or JL) on an Applio 400 US machine (Toshiba Medical Systems, Tochigi, Japan). High-frequency probes were used: a 12-Mhz probe for knee examination and an 18-MHz probe for ankle and foot examination. US examination for the DC sign was performed on the femoro-patellar joints, talo-crural joints, and first metatarsophalangeal joints [17].

### CT data acquisition and image reconstruction

All scans were performed using a single-source CT (Somatom Definition Edge; Siemens, Erlangen, Germany). The patients were positioned feet first in a supine position. Knees and feet were scanned axially in two separate acquisitions performed consecutively on the same day. All scans were performed with the same image protocol, acquisition at 128 × 0.6 mm, and pitch of 0.7. For each body part, two scans were acquired with tube potentials of 80 kV and 140 kV. Depending on the scanned body region, quality reference tube currents ranged between 62 and 260 mAs. Automated attenuation-based tube current modulation was used in all examinations.

Axial images with soft (B30f) and bone (B70f) convolution kernels were reconstructed with a 1-mm slice thickness and an increment of 1 mm. DECT postprocessing was performed by the radiologists with dedicated software (syngo.via VB10B, syngo Dual Energy Gout; Siemens), following the parameters described elsewhere [18]: UH threshold, 150; iodine ratio, 1.4; material definition ratio, 1.25; resolution, 4; air distance, 5; bone distance, 10. Two kinds of images were reconstructed for each body part. First, volume-rendered three-dimensional (3D) images in which urate crystal deposits coded in green were reconstructed with a bone tissue convolution kernel (B70f). These images allowed a straightforward overview of MSU deposits. Second, multiplanar reformations associating images reconstructed with a soft tissue kernel (B30f) and colored images were reconstructed. The aspect of the final fusion images could be changed by modulating the relative percentages of the morphological and colored images from 0 to 100% with a slider.

### Statistical analysis

Statistical analysis was performed using the R software (version 3.4.2). Quantitative variables are expressed as mean and standard deviation, and qualitative variables as number and percentage.

Two-by-two correlations of quantitative variables were assessed by the Spearman correlation coefficient given the absence of normal distribution of values. Tests for nullity of coefficients were performed.

DECT volumes of urate deposition, the number of joints with the DC, and SU levels were compared between the groups of patients presenting with and without the metabolic syndrome using the Mann-Whitney-Wilcoxon test as data were not normal.

The significance level was set at 5%.

The primary endpoint was the Spearman correlation coefficient ρ between DECT MSU volume deposited on the feet and cardiovascular risk.

## Results

0f the 50 patients included, eight were excluded since lipid and glucose levels were not collected. The remaining 42 patients included were predominantly male (40/42) and aged 63.0 ± 13.2 years. Patient characteristics are described in Table 1. Of these 42 patients, 33 had no prior coronary heart disease, peripheral arterial disease, or stroke, and therefore could have their cardiovascular risk scores calculated.

**Table 1:** Population characteristics

Characteristic	
Demographics	
Male (*n* (%))	40 (95.2%)
Age (years)	63 ± 13.2
Gout duration (years)	7.9 ± 9.6
Familial gout (*n* (%))	7 (16.7%)
Number of flares per year	4.1 ± 6.6
Alcohol consumption (g/day)	15 ± 20.7
Creatinine clearance (ml/min)	80.0 ± 31.1
Cardiovascular risk factors	
Body mass index (kg/m^2^)	30.2 ± 5.7
Total cholesterol (mg/dL)	177 ± 63
HDL cholesterol (mg/dL)	44 ± 13
LDL cholesterol (mg/dL)	97 ± 43
Triglycerides (mg/dL)	207 ± 393
Systolic blood pressure (mmHg)	132.8 ± 13.7
Diastolic blood pressure (mmHg)	77 ± 9.9
Smoker (*n* (%))	6 (14.3%)
Metabolic syndrome (*n* (%))	28 (66.7%)
Cardiovascular comorbidities	
Coronary heart disease (*n* (%))	6 (14.3%)
Peripheral arterial disease (*n* (%))	3 (7.1%)
Stroke (*n* (%))	3 (7.1%)
Diabetes mellitus (*n* (%))	15 (35.7%)
Cardiovascular risk assessment	
ACC/AHA 10-year risk (%)	21 ± 14
Framingham 10-year general cardiovascular risk (%)	22 ± 14
Framingham 10-year coronary risk (%)	15 ± 11
Urate burden	
Serum urate (mg/dL)	8.1 ± 2.3
Subcutaneous (clinical) tophi (*n* (%))	12 (28.6%)
Ultrasound tophus (*n* (%))	29 (69%)
Ultrasound tophus volume (cm^3^)	1.1 ± 1.4
At least one joint with the double contour sign (*n* (%))	41 (97.6%)
DECT MSU volume knees (cm^3^) (*n* = 39)	1.9 ± 4.6
DECT MSU volume feet (cm^3^) (*n* = 41)	2.7 ± 6.7
DECT MSU volume knees + feet (cm^3^) (*n* = 38)	4.7 ± 10.8
Ongoing drugs	
Diuretics (*n* (%))	11 (26.2%)
Antidiabetic treatment (*n* (%))	15 (35.7%)
Hypolipidemic treatment (*n* (%))	16 (38.1%)
Treatment for high blood pressure (*n* (%))	25 (59.5%)

*ACC/AHA* American College of Cardiology/American Heart Association, *DECT* dual-energy computed tomography, *HDL* high-density lipoprotein, *LDL* low-density lipoprotein, *MSU* monosodium urate

**Fig. 1:**
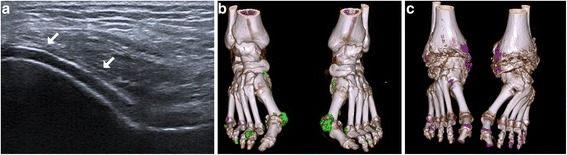
Imaging of monosodium urate crystal deposition. **a** Double contour sign of intra-articular cartilage deposition of the femoro-patellar joint (arrows), (**b**) large (volume 5.39 cm^3^) and (**c**) small (volume 0.02 cm^3^) soft tissue volumes of deposits on the feet visualized with dual-energy computed tomography

Overall, 29/42 had at least one US tophus of 1.1 ± 1.4 cm^3^. Patients presented with 2.2 ± 1.0 joints with the DC sign out of 6 (median 2, interquartile range (IQR) 2–3). The volume of MSU deposits with DECT was 2.7 ± 6.7 cm^3^ for the feet (median 0.7 cm^3^, IQR 0.1–2.2) and 1.9 ± 4.6 cm^3^ for the knees (median 0.2 cm^3^, range 0–1.2) (Fig. 1). Correlations between SU levels and DECT volumes of MSU deposits of the knees, feet, and knees + feet were weak (ρ = 0.28, 0.20, and 0.23, respectively). Overall, 28/42 patients presented with the metabolic syndrome and the average 10-year coronary event or stroke risk according to the ACC/AHA (*n* = 33) was high (21 ± 15%).

**Fig. 2:**
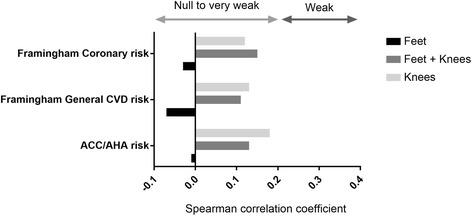
Correlation between the dual-energy computed tomography (DECT) volumes of monosodium urate deposition and the assessment of the risk of coronary heart disease or stroke according to the American College of Cardiology/American Heart Association (ACC/AHA), and the assessment of the Framingham coronary heart disease and general cardiovascular disease risks

**Fig. 3:**
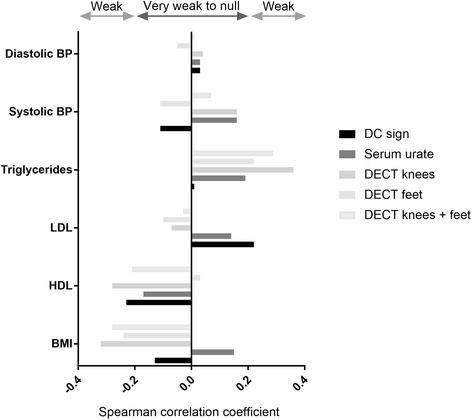
Correlation between the urate burden and individual cardiovascular risk factors. BMI, body mass index; BP, blood pressure; DC, double contour; DECT, dual-energy computed tomography; HDL, high-density lipoprotein; LDL, low-density lipoprotein

Correlations between DECT volumes of MSU deposits in the knees, feet, and knees + feet and cardiovascular risk according to the ACC/AHA were very poor, with ρ = 0.18, −0.01, and 0.13, respectively (Fig. 2), and did not differ significantly from zero (*p* > 0.05). The was no correlation between the number of joints with the DC sign and cardiovascular risk (ρ = −0.07) and the correlation was very poor with SU levels (ρ = 0.15). DECT MSU deposit volume was similar in patients with and without metabolic syndrome (*p* = 0.29) (Table 2). Correlations between the urate burden assessed by SU levels, the number of joints with the DC sign, and DECT volumes of MSU deposition and individual cardiovascular risk factors are weak to null, and are shown in Fig. 3.

**Table 2:** Comparison of dual-energy computed tomography (DECT) volumes of monosodium urate deposition, number of joints presenting with the double contour (DC) sign, and serum urate levels depending on whether the metabolic syndrome is present or not

	Total population (*n* = 42)	No metabolic syndrome (*n* = 14)	Metabolic syndrome (*n* = 28)	*p* value
Number of joints with DC sign	2 (2–3)	2 (1–3)	2 (2–3)	0.65
DECT volume knees (mm^3^)	0.2 (0–1)	0.4 (0–0.6)	0.1 (0–1.2)	0.91
DECT volume feet (mm^3^)	0.5 (0.1–1.8)	0.8 (0.3–1.9)	0.4 (0–1.3)	0.46
DECT volume knees + feet (mm^3^)	0.8 (0.2–2.8)	1.2 (0.6–2.2)	0.4 (0.1–2.3)	0.29
Serum urate level (mg/dL)	7.6 (6.8–9.4)	7.6 (7.2–8.8)	7.5 (6.0–9.4)	0.63

As the Mann-Whitney-Wilcoxon test was used (no normality), data are expressed as median (interquartile range)

## Discussion

This study found no, or only very weak, correlation between levels of overall cardiovascular risk and the extent of urate burden in the knees and feet measured using DECT and US. Urate burden was not associated with the prevalence of the metabolic syndrome. Some weak correlations were established between individual components of cardiovascular risk, notably serum triglyceride levels.

Correlations between urate burden and cardiovascular risk were particularly weak when considering measurements of the feet only. These results are in direct contrast with the conclusions recently made by Lee et al. from their retrospective study [19], considering that there was a correlation between total DECT urate volume of the feet and both the 10-year Framingham risk for cardiovascular disease and prevalence of the metabolic syndrome [19]. Explanations for these discrepancies include biases inherent to retrospective studies that may have affected the study from Lee et al., both the fact that no mention was made on the exclusion of patients with prior major cardiovascular factors for whom cardiovascular risk scores are not applicable, and also potential differences between gout and cardiovascular comorbidities across populations [20, 21], in this case of European and Asian origin. Furthermore, when looking directly at the numbers, the Spearman correlation coefficient for volumes of urate deposition of the feet and the 10-year Framingham risk score for general cardiovascular disease was −0.07 (very weakly negatively correlated to no correlation) in our study but only 0.22 (weak correlation) in the study by Lee et al. [19].

Despite a known higher prevalence of the metabolic syndrome in the population of gout patients, it does not seem to be related to the extent of urate burden [22]. This result is consistent with previous results from the study by Lee et al. in which the metabolic syndrome was not associated with the volume of urate deposition in multivariate analysis [19]. Surprisingly, despite a known trend of increasing SU levels with BMI [23], all volumes of MSU deposits measured with DECT were negatively weakly correlated to the BMI. A technical explanation could be that it is known that visceral fat increases noise in the 80 kV images [24]; this remains to be shown for peripheral joints. A weak negative correlation of the prevalence of the DC sign with the BMI was also found, which could also be explained by the difficulty of observing joints with US with greater surrounding adipose tissue. However, so far, no study has reported difficulties in the search for the DC sign in peripheral joints of obese patients.

Cholesterol abnormalities (increased LDL and decreased HDL cholesterol) are weakly associated with joint MSU deposition. Our study found a weak association of HDL levels with intra-articular MSU deposition assessed with the US DC sign, urate burden of the knees with DECT, and SU levels, and a weak association between LDL levels and the DC sign only. These weak or very weak correlations are consistent with the results from the third NHANES which showed an increased prevalence of low HDL in gout patients, but to a far lesser extent than the high prevalence of hypertriglyceridemia [22]. It is unclear whether these correlations are only due to the same correlations between LDL and HDL levels with SU that are similar.

An increase in triglyceride levels in gout is weakly associated with an increased MSU soft tissue deposition measured with DECT. Genetic associations showed that mutations in the apolipoprotein gene cluster play a causal role in gout, even after adjustment for lipid and SU levels [21]. Apolipoprotein plays a central role in lipid metabolism and, notably, in the transport of triglycerides. A study using Mendelian randomization has previously shown the causal role of triglycerides in raising SU levels in men, but the reverse was not true [25]. Elevations of very low-density lipoprotein (VLDL)-triglycerides may be implicated in the transition between asymptomatic hyperuricemia and gout [20]. One of the explanations for the association between lipid modifications and gout would be that lipids are of importance in the coating of MSU crystals and that modifications of this coating has implications on the inflammatory response to the presence of these crystals [26]. The correlation between triglyceride levels and MSU burden found in our study suggests that not only do triglycerides increase circulating urate but are also possibly involved in MSU deposition itself.

We acknowledge that this study presents with some limitations. The first one is sample size, which limited our ability to establish the precise level of the correlations studied. Second, the fact that most gout patients had significant cardiovascular risk as shown in other studies made identifying factors able to discriminate between levels of risk difficult [6]. Nonetheless, given the fact that all correlations found were null to weak, it seems improbable that a strong correlation has been missed using our methodology. The third limitation is inherent to the cross-sectional nature of the study itself as it cannot establish a correlation between urate burden and prevalence of cardiovascular events. The present study can only establish whether there is a link between MSU burden and cardiovascular risk factors. A longitudinal study is necessary to explore if the MSU burden is an independent risk for cardiovascular events.

## Conclusions

The present study demonstrates that, while the quantity of urate burden is involved in the association of gout with increased cardiovascular events, it does not seem to be through an overall increase in traditional cardiovascular risk factors. The extent of MSU burden does not increase the estimated risk of cardiovascular events and, thus, quantifying the MSU burden is not a surrogate marker for traditional cardiovascular risk assessment of gout patients naive of urate lowering therapy.

## Abbreviations

ACC: American College of Cardiology
ACR: American College of Rheumatology
AHA: American Heart Association
BMI: Body mass index
BP: Blood pressure
DC: Double contour
DECT: Dual-energy computed tomography
eGFR: Estimated glomerular filtration rate
EULAR: European League Against Rheumatism
HDL: High-density lipoprotein
LDL: Low-density lipoprotein
MSU: Monosodium urate
SU: Serum urate
US: Ultrasonography
